# B-Cell Responses to Intramuscular Administration of a Bivalent Virus-Like Particle Human Norovirus Vaccine

**DOI:** 10.1128/CVI.00571-16

**Published:** 2017-05-05

**Authors:** Sasirekha Ramani, Frederick H. Neill, Jennifer Ferreira, John J. Treanor, Sharon E. Frey, David J. Topham, Robert R. Goodwin, Astrid Borkowski, Frank Baehner, Paul M. Mendelman, Mary K. Estes, Robert L. Atmar

**Affiliations:** a Baylor College of Medicine, Houston, Texas, USA; b The Emmes Corp., Rockville, Maryland, USA; c University of Rochester School of Medicine and Dentistry, Rochester, New York, USA; d Saint Louis University, School of Medicine, St. Louis, Missouri, USA; e Takeda Vaccines, Inc., Deerfield, Illinois, USA; f Takeda Pharmaceuticals International AG, Zurich, Switzerland; Vanderbilt University Medical Center

**Keywords:** norovirus, vaccine, VLP, immunity, antibody-secreting cells, memory B cells, immune response

## Abstract

Human noroviruses (HuNoVs) are a leading cause of acute gastroenteritis worldwide. A virus-like particle (VLP) candidate vaccine induces the production of serum histo-blood group antigen (HBGA)-blocking antibodies, the first identified correlate of protection from HuNoV gastroenteritis. Recently, virus-specific IgG memory B cells were identified to be another potential correlate of protection against HuNoV gastroenteritis. We assessed B-cell responses following intramuscular administration of a bivalent (genogroup I, genotype 1 [GI.1]/genogroup II, genotype 4 [GII.4]) VLP vaccine using protocols identical to those used to evaluate cellular immunity following experimental GI.1 HuNoV infection. The kinetics and magnitude of cellular immunity to G1.1 infection were compared to those after VLP vaccination. Intramuscular immunization with the bivalent VLP vaccine induced the production of antibody-secreting cells (ASCs) and memory B cells. ASC responses peaked at day 7 after the first dose of vaccine and returned to nearly baseline levels by day 28. Minimal increases in ASCs were seen after a second vaccine dose at day 28. Antigen-specific IgG memory B cells persisted at day 180 postvaccination for both GI.1 and GII.4 VLPs. The overall trends in B-cell responses to vaccination were similar to the trends in the responses to infection, where there was a greater bias of an ASC response toward IgA and a memory B-cell response to IgG. The magnitude of the ASC and memory B-cell responses to the GI.1 VLP component of the vaccine was also comparable to that of the responses following GI.1 infection. The production of IgG memory B cells and persistence at day 180 is a key finding and underscores the need for future studies to determine if IgG memory B cells are a correlate of protection following vaccination. (This study has been registered at ClinicalTrials.gov under registration no. NCT01168401.)

## INTRODUCTION

Noroviruses are positive-sense, single-stranded RNA viruses belonging to the family Caliciviridae (1). The genus Norovirus is classified into at least 7 genogroups (genogroup I [GI] to GVII), and viruses in GI, GII, and GIV are known to cause infections in humans (2). Each genogroup is further subdivided into genotypes on the basis of phylogenetic analysis of the major capsid protein VP1. Over 31 genotypes of human noroviruses (HuNoV) have been reported, including 9 GI genotypes and 22 GII genotypes (2). The first isolated HuNoV, Norwalk virus (NV), belongs to genogroup I, genotype 1 (GI.1), while the majority of HuNoV outbreaks worldwide are caused by genogroup II, genotype 4 (GII.4), strains (3).

Globally, HuNoV infections account for nearly 18% of all cases of acute gastroenteritis. Higher prevalence rates are seen in the community (24%) and in outpatient settings (20%) than in inpatient settings (17%) (4). In countries where rotavirus vaccines are effective, HuNoVs have replaced rotavirus as the most common cause of pediatric viral gastroenteritis (5, 6). In the United States, HuNoV infections are the leading cause of sporadic and epidemic gastroenteritis across all age groups, resulting in 19 million to 21 million total cases of illness, 1.7 million to 1.9 million outpatient visits, 400,000 emergency department visits, 56,000 to 71,000 hospitalizations, and 570 to 800 deaths annually (7). Globally, HuNoV costs over $4.2 billion in direct health care expenditures, which is only a fraction of the estimated total of $60.3 billion in societal costs annually (8).

The significant public health and economic burden of HuNoV gastroenteritis underscores the need for safe and effective vaccines. The *in vitro* expression of HuNoV capsid proteins results in the self-assembly of virus-like particles (VLPs) that are morphologically and antigenically similar to the infectious virus (9, 10). VLPs produced in a recombinant baculovirus expression system have been evaluated as vaccine candidates in preclinical and clinical studies (11). Oral, intranasal, and intramuscular immunizations with HuNoV VLPs were found to be safe and immunogenic and were efficacious in proof-of-principle clinical efficacy studies (12–16). Monovalent and bivalent vaccine formulations containing GI.1 VLPs alone and GI.1 and GII.4 VLPs, respectively, have been tested. Intranasal and intramuscular immunization results in the induction of functional antibodies in serum that block the binding of VLPs to cell attachment factors called histo-blood group antigens (HBGAs) (12, 16). HBGA-blocking antibodies were the first recognized correlate of protection from HuNoV gastroenteritis. Identified initially from human volunteer experimental challenge studies, HBGA-blocking antibodies were confirmed to be a correlate of protection in clinical trials with VLP vaccines (12, 13, 17).

B-cell responses following experimental infection with NV were recently evaluated. Assessment of pre- and postchallenge levels of IgA and IgG antibody-secreting cells (ASC) and total and virus-specific IgA and IgG memory B cells revealed that the preinfection levels of virus-specific IgG memory B cells correlate with protection from acute gastroenteritis (18). While the induction of mucosal homing ASCs following intranasal and intramuscular immunization with VLP vaccines was demonstrated previously (14, 19, 20), memory B-cell responses were measured only following intranasal immunization with a monovalent GI.1 vaccine (20). Further, the approaches used to evaluate ASC and memory B-cell responses in previous HuNoV vaccine studies differed from those used in NV experimental infection studies. In this study, we determined the B-cell (ASC and memory B-cell) responses to intramuscular administration of bivalent (GI.1 and GII.4) VLP vaccine formulations. The use of protocols identical to those used to evaluate B-cell responses following experimental human infection with GI.1 virus allowed us to compare the kinetics and the magnitude of the B-cell responses between G1.1 infection and vaccination with GI.1 VLPs.

## RESULTS

### IgA and IgG antibody-secreting cells.

IgA and IgG ASC responses to GI.1 and GII.4 (consensus) VLPs were measured prior to each vaccine dose (days 0 and 28) and at 7 days postvaccination for both doses (days 7 and 35). Peak IgA and IgG ASC responses were seen on day 7 following the first vaccine dose for all dosage groups (Table 1 and Table 2, respectively; see also Fig. S1 in the supplemental material). All vaccine recipients (100%) showed ≥4-fold rises in the levels of ASCs from prevaccination levels at this time point. IgA and IgG ASC responses were reduced dramatically by day 28, prior to administration of the second dose. Although the second dose of vaccination induced increases in ASC levels, the rises in titers were modest compared to those after the first vaccine dose. Significant differences in peak IgA ASC levels and geometric mean fold rises from the baseline were observed between the different vaccine dose groups for both the GI.1 and GII.4 VLPs (*P* < 0.05). For IgG ASCs, significant differences in peak responses between the different dose groups were observed only for GII.4 VLPs (*P* < 0.01). The magnitude of the ASC response to GI.1 VLPs was higher than that to GII.4 VLPs for both IgA (Fig. S2A) and IgG (Fig. S2B). For IgA ASCs, the responses to GI.1 were significantly higher at days 7 and 35 after vaccination (*P* < 0.001 and *P* = 0.034, respectively), while for IgG ASCs, significant differences were seen on days 7 and 28 (*P* < 0.001) but not on day 35 (*P* = 0.12). The magnitude of the IgA ASC response postvaccination was higher than that of the IgG response for both VLPs. For GI.1 VLPs, the IgA ASC response was significantly higher than the IgG ASC response at days 7, 28, and 35 (*P* < 0.001 for all three time points), while significant differences were seen on days 7 and 28 postvaccination for GII.4 VLPs (*P* < 0.001 and *P* = 0.003, respectively).

**TABLE 1:** IgA ASC responses to GI.1 and GII.4 VLPs

Treatment group	No. of participants	Day	GI.1			GII.4		
			Geometric mean^*a*^ (95% CI^*b*^)	Geometric mean fold rise (95% CI)	% with 4-fold rise (95% CI)	Geometric mean (95% CI)	Geometric mean fold rise (95% CI)	% with 4-fold rise (95% CI)
Placebo	8	0	8			8		
	8	7	15 (4, 59)	1.8 (0.4, 7.4)	12.5 (0.3, 52.7)	20 (2, 163)	2.4 (0.3, 20.3)	12.5 (0.3, 52.7)
	8	28	8	1.0	0.0 (0.0, 36.9)	8	1.0	0.0 (0.0, 36.9)
	7	35	9 (7, 11)	1.1 (0.9, 1.4)	0.0 (0.0, 41.0)	11 (5, 23)	1.4 (0.6, 2.9)	14.3 (0.4, 57.9)
5/5 μg	10	0	8			8		
	10	7	9,545 (5,039, 18,077)	1,193 (629.9, 2,260)	100.0 (69.2, 100.0)	2,170 (863, 5,458)	271.2 (107.8, 682.2)	100.0 (69.2, 100.0)
	9	28	9 (7, 11)	1.1 (0.9, 1.4)	0.0 (0.0, 33.6)	9 (7, 11)	1.1 (0.9, 1.3)	0.0 (0.0, 33.6)
	9	35	46 (18, 121)	5.8 (2.2, 15.1)	55.6 (21.2, 86.3)	126 (37, 429)	15.7 (4.6, 53.7)	77.8 (40.0, 97.2)
15/15 μg	9	0	8			8		
	9	7	3,367 (968, 11,705)	420.9 (121.1, 1,463)	100.0 (66.4, 100.0)	886 (381, 2,057)	110.7 (47.7, 257.1)	100.0 (66.4, 100.0)
	8	28	12 (6, 21)	1.5 (0.8, 2.7)	12.5 (0.3, 52.7)	10 (6, 15)	1.2 (0.8, 1.9)	12.5 (0.3, 52.7)
	7	35	32 (13, 79)	4.0 (1.6, 9.9)	14.3 (0.4, 57.9)	46 (9, 244)	5.8 (1.1, 30.5)	57.1 (18.4, 90.1)
50/50 μg	9	0	9 (7, 10)			8		
	9	7	9,262 (2,285, 37,541)	1,072 (243.7, 4,714)	100.0 (66.4, 100.0)	2,806 (569, 13,825)	350.7 (71.2, 1,728)	100.0 (66.4, 100.0)
	8	28	19 (8, 41)	2.1 (1.0, 4.4)	25.0 (3.2, 65.1)	8	1.0	0.0 (0.0, 36.9)
	8	35	30 (13, 67)	3.4 (1.5, 8.0)	37.5 (8.5, 75.5)	26 (12, 56)	3.2 (1.5, 7.0)	50.0 (15.7, 84.3)
150/150 μg	6	0	8			8		
	6	7	36,303 (12,580, 104,758)	4,538 (1,573, 13,095)	100.0 (54.1, 100.0)	15,374 (3,360, 70,336)	1,922 (420.0, 8,792)	100.0 (54.1, 100.0)
	6	28	18 (6, 56)	2.2 (0.7, 7.0)	16.7 (0.4, 64.1)	27 (10, 68)	3.3 (1.3, 8.5)	16.7 (0.4, 64.1)
	5	35	90 (32, 252)	11.2 (4.0, 31.4)	100.0 (47.8, 100.0)	327 (128, 836)	40.9 (16.0, 104.4)	100.0 (47.8, 100.0)

a The geometric mean number of ASCs per million PBMCs is given for each study group.

b CI, confidence interval.

**TABLE 2:** IgG ASC responses to GI.1 and GII.4 VLPs

Treatment group	No. of participants	Day	GI.1			GII.4		
			Geometric mean^*a*^ (95% CI^*b*^)	Geometric mean fold rise (95% CI)	% with 4-fold rise (95% CI)	Geometric mean (95% CI)	Geometric mean fold rise (95% CI)	% with 4-fold rise (95% CI)
Placebo	8	0	8			8		
	8	7	13 (4, 38)	1.6 (0.5, 4.7)	12.5 (0.3, 52.7)	17 (3, 102)	2.1 (0.4, 12.8)	12.5 (0.3, 52.7)
	8	28	8	1.0	0.0 (0.0, 36.9)	9 (7, 12)	1.1 (0.9, 1.4)	0.0 (0.0, 36.9)
	7	35	8	1.0	0.0 (0.0, 41.0)	13 (4, 47)	1.7 (0.5, 5.8)	14.3 (0.4, 57.9)
5/5 μg	10	0	9 (7, 12)			9 (7, 12)		
	10	7	2,598 (1,010, 6,683)	289.8 (102.9, 816.3)	100.0 (69.2, 100.0)	758 (336, 1,712)	84.2 (39.0, 181.7)	100.0 (69.2, 100.0)
	9	28	40 (18, 91)	4.4 (1.6, 12.2)	77.8 (40.0, 97.2)	11 (7, 19)	1.2 (0.6, 2.4)	22.2 (2.8, 60.0)
	9	35	131 (73, 235)	14.4 (6.8, 30.6)	88.9 (51.8, 99.7)	105 (56, 195)	11.5 (5.0, 26.3)	88.9 (51.8, 99.7)
15/15 μg	9	0	8			8		
	9	7	2,538 (923, 6,981)	317.3 (115.4, 872.6)	100.0 (66.4, 100.0)	610 (288, 1,292)	76.3 (36.0, 161.5)	100.0 (66.4, 100.0)
	8	28	58 (17, 199)	7.2 (2.1, 24.9)	75.0 (34.9, 96.8)	30 (11, 83)	3.8 (1.4, 10.3)	50.0 (15.7, 84.3)
	7	35	192 (75, 493)	24.0 (9.4, 61.6)	100.0 (59.0, 100.0)	159 (73, 346)	19.9 (9.2, 43.2)	100.0 (59.0, 100.0)
50/50 μg	9	0	8			8		
	9	7	4,578 (1,296, 16,170)	572.2 (162.0, 2,021)	100.0 (66.4, 100.0)	956 (253, 3,610)	119.5 (31.6, 451.3)	100.0 (66.4, 100.0)
	8	28	66 (34, 129)	8.2 (4.2, 16.1)	75.0 (34.9, 96.8)	29 (11, 76)	3.7 (1.4, 9.6)	25.0 (3.2, 65.1)
	8	35	156 (87, 278)	19.5 (10.9, 34.7)	100.0 (63.1, 100.0)	83 (40, 174)	10.4 (5.0, 21.8)	87.5 (47.3, 99.7)
150/150 μg	6	0	12 (4, 37)			8		
	6	7	10,239 (3,442, 30,459)	831.4 (143.8, 4,807)	100.0 (54.1, 100.0)	7,245 (1,329, 39,488)	905.6 (166.1, 4,936)	100.0 (54.1, 100.0)
	6	28	81 (28, 229)	6.5 (1.2, 35.1)	66.7 (22.3, 95.7)	23 (6, 89)	2.9 (0.8, 11.1)	33.3 (4.3, 77.7)
	5	35	198 (17, 2,337)	14.7 (0.3, 669.0)	80.0 (28.4, 99.5)	199 (112, 353)	24.9 (14.0, 44.2)	100.0 (47.8, 100.0)

a The geometric mean number of ASCs per million PBMCs is given for each study group.

b CI, confidence interval.

**FIG 1:**
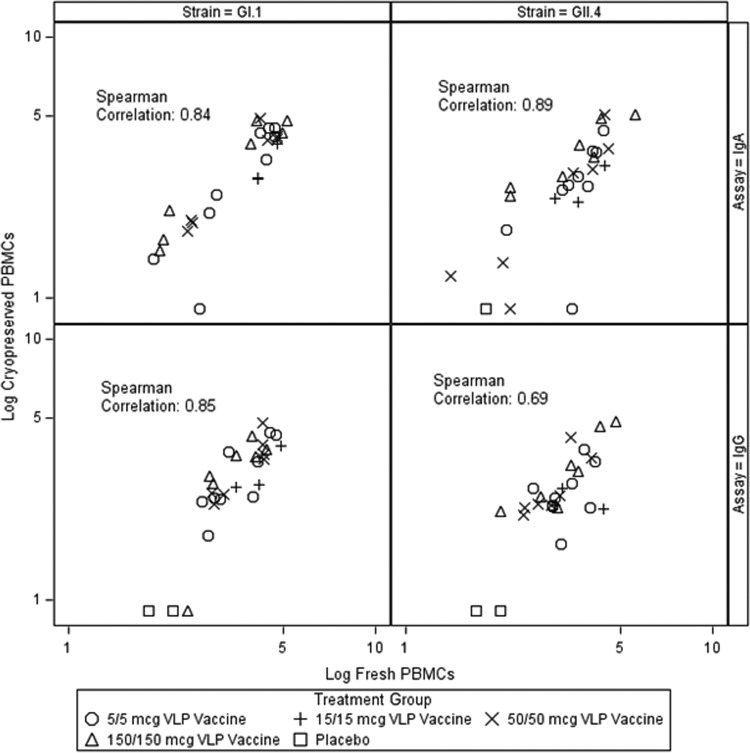
Correlation of IgA and IgG ASC responses obtained using fresh and cryopreserved PBMCs. ASC responses at days 7 and 35 (7 days after each vaccine dose) were compared. A strong correlation between the results obtained using fresh and cryopreserved PBMCs was seen, as indicated by the Spearman correlation coefficient given in each panel. Results for ASC assays using fresh PBMCs were published previously (19).

While the results for ASCs reported in this study were obtained using cryopreserved peripheral blood mononuclear cells (PBMCs), ASC levels were previously determined using fresh PBMCs from a subset of subjects in a study (19), allowing the comparison of results. IgA and IgG ASC assays were previously carried out using fresh PBMCs from 29 samples collected at the University of Rochester (19). The overall trends in the results were similar, with the peak response being at day 7 after the first vaccine dose and with a greater bias toward IgA ASCs and a stronger response to GI.1 VLPs than GII.4 VLPs being seen. Strong correlations between the results of all assays with ASCs obtained using cryopreserved PBMCs and fresh PBMCs were observed (Fig. 1).

### IgA and IgG memory B cells.

IgA and IgG memory B-cell responses (Tables 3 and 4, respectively, and Fig. S3) to GI.1 and GII.4 VLPs were measured prior to each vaccine dose (days 0 and 28) and at days 56 and 180 postvaccination. The percentages of VLP-specific memory B cells per 0.5 million total memory B cells were compared between groups. There were no significant differences in the memory B-cell responses across the different vaccine dose groups. However, unlike ASCs, the responses of which peaked at day 7 after the first dose of vaccine, the peak memory B-cell response varied between the two VLPs and between the IgA and IgG assays. The magnitude of the IgA memory B-cell response to GI.1 VLPs was significantly higher than that to GII.4 VLPs at day 28 (*P* = 0.033) (Fig. S4A) but not at day 56 after the first dose of vaccine (Fig. S4B). The IgG memory B-cell responses to GI.1 VLPs were significantly higher than the responses to GII.4 VLPs at all time points postvaccination (*P* = <0.05) (data for days 28 and 56 are shown in Fig. S4C and D, respectively). In contrast to the ASC responses, the magnitudes of the IgG memory B-cell responses were significantly higher (*P* < 0.001) than those of the IgA responses for both VLPs at all time points after vaccination. The percentage of VLP-specific IgA memory B cells returned to near-baseline values by day 180 postvaccination, while the percentage of VLP-specific IgG memory B cells persisted at this time point.

**TABLE 3:** IgA memory B-cell responses to GI.1 and GII.4 VLPs

Treatment group	Day	GI.1					GII.4				
		No. of participants	Geometric mean^*a*^ (95% CI^*b*^)	No. of participants^*c*^	Geometric mean fold rise (95% CI)	% with 4-fold rise (95% CI)	No. of participants	Geometric mean (95% CI)	No. of participants	Geometric mean fold rise (95% CI)	% with 4-fold rise (95% CI)
Placebo	0	4	0.29 (0.11, 0.74)				4	0.30 (0.11, 0.80)			
	28	4	0.22 (0.05, 1.01)	4	0.8 (0.2, 3.4)	0.0 (0.0, 60.2)	6	0.43 (0.12, 1.55)	4	1.0 (0.5, 2.1)	0.0 (0.0, 60.2)
	56	3	0.42 (0.09, 2.03)	3	1.2 (0.5, 2.8)	0.0 (0.0, 70.8)	7	0.36 (0.18, 0.73)	4	1.0 (0.4, 2.5)	0.0 (0.0, 60.2)
	180	2	0.21 (0.00, 136.68)	2	1.0 (0.0, 51.6)	0.0 (0.0, 84.2)	4	0.32 (0.22, 0.46)	3	1.0 (0.2, 4.7)	0.0 (0.0, 70.8)
5/5 μg	0	5	0.32 (0.11, 0.90)				5	0.26 (0.17, 0.41)			
	28	9	0.81 (0.55, 1.20)	5	3.2 (0.8, 13.4)	40.0 (5.3, 85.3)	9	0.34 (0.21, 0.52)	5	1.6 (1.0, 2.4)	0.0 (0.0, 52.2)
	56	8	0.65 (0.33, 1.26)	4	2.6 (0.6, 11.5)	25.0 (0.6, 80.6)	5	0.36 (0.15, 0.83)	3	1.7 (0.7, 4.4)	0.0 (0.0, 70.8)
	180	7	0.38 (0.16, 0.91)	5	1.8 (0.5, 5.8)	20.0 (0.5, 71.6)	6	0.34 (0.12, 0.94)	4	1.0 (0.6, 1.6)	0.0 (0.0, 60.2)
15/15 μg	0	2	0.13 (0.05, 0.29)				7	0.26 (0.15, 0.45)			
	28	9	0.58 (0.25, 1.35)	2	4.7 (0.0, 6,774.1)	50.0 (1.3, 98.7)	9	0.30 (0.15, 0.62)	6	1.5 (0.7, 3.5)	16.7 (0.4, 64.1)
	56	7	0.58 (0.24, 1.36)	2	2.7 (0.0, 9,311.2)	50.0 (1.3, 98.7)	6	0.41 (0.22, 0.77)	4	1.5 (0.6, 3.3)	0.0 (0.0, 60.2)
	180	8	0.36 (0.22, 0.60)	2	2.5 (0.0, 15,017.8)	50.0 (1.3, 98.7)	5	0.38 (0.19, 0.73)	4	1.1 (0.6, 1.8)	0.0 (0.0, 60.2)
50/50 μg	0	2	0.10 (0.02, 0.40)				2	0.30 (0.01, 8.59)			
	28	8	0.66 (0.32, 1.35)	2	8.7 (0.0, 28,646.0)	100.0 (15.8, 100.0)	5	0.69 (0.17, 2.73)	2	5.9 (0.0, 23034,090)	50.0 (1.3, 98.7)
	56	6	0.37 (0.13, 1.08)	2	3.9 (0.0, 671.0)	50.0 (1.3, 98.7)	4	0.50 (0.07, 3.48)	2	4.4 (0.0, 184,166.5)	50.0 (1.3, 98.7)
	180	5	0.33 (0.10, 1.11)	1	3.2	0.0 (0.0, 97.5)	5	0.27 (0.07, 0.97)	2	2.3 (0.0, 28,207.5)	50.0 (1.3, 98.7)
150/150 μg	0	1	0.3				4	0.45 (0.21, 0.94)			
	28	8	0.62 (0.33, 1.17)	1	5.8	100.0 (2.5, 100.0)	7	0.69 (0.26, 1.86)	4	2.7 (0.5, 15.1)	50.0 (6.8, 93.2)
	56	8	0.29 (0.15, 0.59)	1	3.5	0.0 (0.0, 97.5)	8	0.51 (0.21, 1.23)	4	1.2 (0.2, 8.9)	25.0 (0.6, 80.6)
	180	8	0.31 (0.19, 0.51)	1	2.1	0.0 (0.0, 97.5)	7	0.56 (0.28, 1.11)	4	1.1 (0.5, 2.3)	0.0 (0.0, 60.2)

a The geometric mean percentage of VLP-specific memory B cells per 5 × 10^5^ total memory B cells is given for each study group.

b CI, confidence interval.

c The second column headed “No. of participants” within each genogroup applies to fold change calculations. This number varied based on the number of participants for whom geometric mean numbers of memory B cells were available at baseline.

**TABLE 4:** IgG memory B-cell responses to GI.1 and GII.4 VLPs

Treatment group	Day	GI.1					GII.4				
		No. of participants	Geometric mean^*a*^ (95% CI^*b*^)	No. of participants^*c*^	Geometric mean fold rise (95% CI)	% with 4-fold rise (95% CI)	No. of participants	Geometric mean (95% CI)	No. of participants	Geometric mean fold rise (95% CI)	% with 4-fold rise (95% CI)
Placebo	0	1	0.6				1	0.25			
	28	1	0.28	1	0.5	0.0 (0.0, 97.5)	2	0.19 (0.00, 1,952.85)	1	1.6	0.0 (0.0, 97.5)
	56	1	0.15	1	0.3	0.0 (0.0, 97.5)	2	0.34 (0.00, 211.37)	1	0.8	0.0 (0.0, 97.5)
	180	1	0.09				2	0.42 (0.00, 101,101.8)	1	0.6	0.0 (0.0, 97.5)
5/5 μg	0	4	0.39 (0.08, 1.86)				7	0.21 (0.13, 0.35)			
	28	9	5.11 (2.99, 8.73)	4	7.9 (2.1, 29.2)	75.0 (19.4, 99.4)	9	1.58 (0.61, 4.07)	6	7.8 (2.3, 26.3)	83.3 (35.9, 99.6)
	56	8	6.80 (2.95, 15.68)	3	7.9 (0.2, 310.3)	66.7 (9.4, 99.2)	8	1.46 (0.41, 5.23)	5	8.3 (1.6, 41.4)	80.0 (28.4, 99.5)
	180	8	4.59 (1.96, 10.74)	4	5.8 (0.7, 46.6)	75.0 (19.4, 99.4)	9	1.07 (0.45, 2.56)	7	4.6 (1.6, 13.4)	57.1 (18.4, 90.1)
15/15 μg	0	5	0.37 (0.17, 0.84)				6	0.43 (0.13, 1.38)			
	28	7	4.63 (2.57, 8.34)	4	9.8 (2.4, 40.3)	100.0 (39.8, 100.0)	8	2.11 (0.77, 5.77)	5	4.3 (2.1, 8.7)	40.0 (5.3, 85.3)
	56	5	5.95 (2.09, 16.98)	3	12.7 (1.9, 86.3)	100.0 (29.2, 100.0)	6	2.19 (0.63, 7.54)	5	4.5 (2.8, 7.4)	80.0 (28.4, 99.5)
	180	7	5.81 (2.37, 14.20)	4	8.0 (2.0, 31.2)	75.0 (19.4, 99.4)	8	1.33 (0.48, 3.72)	6	2.4 (1.5, 3.7)	16.7 (0.4, 64.1)
50/50 μg	0	2	0.60 (0.00, 22,908.29)				1	0.14			
	28	4	5.03 (0.77, 32.96)	2	20.8 (0.1, 3,574.8)	100.0 (15.8, 100.0)	4	1.36 (0.09, 21.46)	1	4.5	100.0 (2.5, 100.0)
	56	4	6.99 (3.27, 14.93)	2	14.9 (0.1, 3,481.6)	100.0 (15.8, 100.0)	4	2.84 (0.63, 12.89)	1	7.1	100.0 (2.5, 100.0)
	180	4	7.47 (3.23, 17.32)	2	13.3 (0.0, 28768,477)	100.0 (15.8, 100.0)	4	1.63 (0.18, 14.64)	1	1.6	0.0 (0.0, 97.5)
150/150 μg	0	3	0.76 (0.04, 16.30)				5	0.51 (0.11, 2.43)			
	28	7	6.66 (4.47, 9.93)	2	19.1 (0.0, 20,444.4)	100.0 (15.8, 100.0)	7	4.04 (1.21, 13.52)	5	5.9 (2.9, 11.9)	80.0 (28.4, 99.5)
	56	7	7.07 (4.25, 11.77)	3	9.5 (0.4, 215.4)	100.0 (29.2, 100.0)	7	2.94 (0.96, 8.97)	5	4.0 (2.0, 7.9)	60.0 (14.7, 94.7)
	180	7	15.63 (8.42, 29.00)	3	26.1 (0.7, 1,026.9)	100.0 (29.2, 100.0)	7	1.54 (0.52, 4.58)	5	2.3 (0.7, 8.3)	40.0 (5.3, 85.3)

a The geometric mean percentage of VLP-specific memory B cells per 5 × 10^5^ total memory B cells is given for each study group.

b CI, confidence interval.

c The second column headed “No. of participants” within each genogroup applies to fold change calculations. This number varied based on the number of participants for whom geometric mean numbers of memory B cells were available at baseline.

### Comparison of cellular immune responses to infection and vaccination.

**FIG 2:**
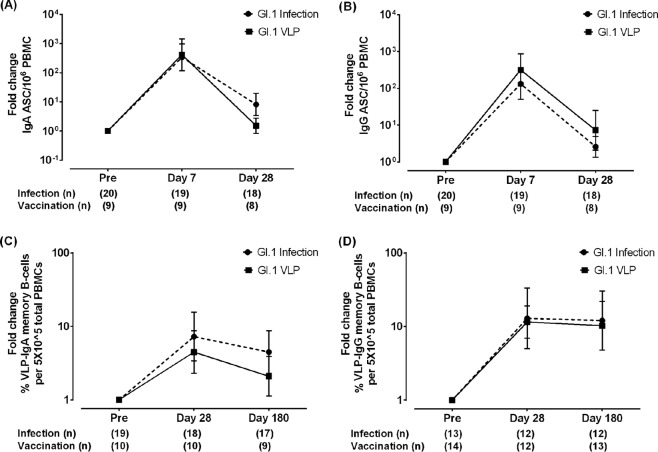
Comparison of cellular immune responses to infection with GI.1 HuNoV and intramuscular immunization with GI.1 VLPs in a bivalent (GI.1/GII.4) VLP vaccine. (A, B) The geometric mean fold changes in the number of IgA ASCs (A) and IgG ASCs (B) at days 7 and 28 postinfection and postvaccination are shown. (C, D) The fold change in the percentage of virus- or VLP-specific memory B cells at days 28 and 180 postinfection and -vaccination was compared for IgA memory B cells (C) and IgG memory B cells (D). Error bars represent 95% confidence intervals.

We previously reported on the ASC and memory B-cell responses following experimental challenge with GI.1 virus, determined using cryopreserved PBMCs and the same protocols described in this study (18). This provided the opportunity to compare cellular immune responses to GI.1 virus infection and vaccination with GI.1 VLPs. For the ASC assays, since significant differences in responses were observed between the different vaccine dose groups, comparisons with infection were carried out using data for the group immunized with the 15-μg vaccine dose that was used in subsequent clinical trials of VLP vaccines (21). Data from the ASC assays were available for infection and vaccination at day 0 and at days 7 and 28 postvaccination or postinfection, and the fold changes from the values at baseline were compared. The ASC responses to both infection and vaccination peaked at day 7, and at this time point, the fold changes in IgA and IgG ASC levels (Fig. 2A and B, respectively) were comparable to those in response to infection with GI.1 virus. ASC levels returned to close to baseline values by day 28 postvaccination. While IgG ASC responses to vaccination remained comparable to those to infection (Fig. 2A), IgA ASC responses dropped to lower levels at day 28 (Fig. 2B).

Data for memory B cells from days 0, 28, and 180 of the infection and vaccination studies were available. Since the number of persons in each dose group with memory B-cell counts above the assay limit of detection was small and there were no significant differences between the different vaccine dose groups, pooled data obtained from all dose groups after vaccination were compared to data obtained after infection. The fold changes in the percentage of GI.1-specific IgA memory B cells were lower following GI.1 vaccination than following infection (Fig. 2C). Interestingly, antigen-specific IgG memory B cells, previously identified to be a correlate of protection against NV gastroenteritis (18), persisted to similar levels at day 180 following both infection and vaccination (Fig. 2D). The fold changes in the percentage of GI.1-specific IgG memory B cells were comparable at day 28 and day 180.

### Correlation between B-cell and serological responses.

Correlation analyses were carried out to determine the relationship between B-cell responses and serological markers of the immune response. We previously demonstrated that following GI.1 infection, peak IgA ASC levels at day 7 correlated with peak serum IgA levels at day 14 (Pearson *r* = 0.86) (18). For vaccine studies, peak IgA responses were seen at day 7 after the first vaccine dose (16), and no significant correlations between serum IgA and ASC levels were observed at this time point. While serological analyses were not carried out at day 14 for the vaccine studies, we determined whether ASC levels correlated with antibody levels at later time points. Significant correlations were seen between GI.1 VLP-specific IgA and IgG ASC concentrations on day 7 and the corresponding serum IgA and IgG antibody responses on day 21 (Spearman *r* = 0.57 and *P* < 0.01 and Spearman *r* = 0.42 and *P* < 0.05, respectively). Interestingly, GII.4 VLP-specific IgA and IgG ASC levels were significantly correlated with serum HBGA-blocking antibody responses at day 21 (Spearman *r* = 0.38 and *P* < 0.05 and Spearman *r* = 0.44 and *P* < 0.01, respectively) and pan-Ig antibody responses (Spearman *r* = 0.43 and *P* < 0.05 and Spearman *r* = 0.55 and *P* < 0.01, respectively) rather than IgA or IgG responses, highlighting the differences in the responses to the two VLPs.

Correlation analyses were also performed to assess the relationship between antigen-specific memory B cells and other immune correlates of protection against HuNoV gastroenteritis, such as serum HBGA-blocking antibody levels and serum IgA levels (22). The number of GI.1 IgA memory B cells prior to vaccination correlated with prevaccination serum HBGA antibody levels (Spearman *r* = 0.42, *P* < 0.01). The number of GI.1 and GII.4 IgG memory B cells prevaccination also correlated with HBGA antibody levels (Spearman *r* = 0.56 and *P* < 0.01 and Spearman *r* = 0.44 and *P* < 0.05, respectively). Since memory B cells are important for long-term immunity, we also evaluated the correlation between memory B cell levels and serological correlates of protection 6 to 12 months postvaccination. The percentages of GI.1- and GII.4-specific IgA memory B cells on day 56 after the first vaccine dose were significantly correlated with the serum titers of genotype-specific IgA on both days 180 and 393 (*P* < 0.05). The percentage of GI.1-specific IgA memory B cells also correlated with serum GI.1-specific HBGA-blocking antibody levels on day 393 (Spearman *r* = 0.42, *P* < 0.05).

## DISCUSSION

Immunogenicity to HuNoV VLP vaccines has been evaluated in a number of preclinical and clinical studies involving different vaccine formulations and routes of immunization. Serological assessments have shown the induction of robust antibody responses. Serum HBGA-blocking antibody and IgA levels were found to correlate with protection from gastroenteritis on challenge with infectious virus following vaccination (12, 23). Recent work on B-cell responses following NV infection showed that the levels of virus-specific IgG memory B cells also correlate with protection from gastroenteritis (18). The persistence of IgG memory B cells on day 180 postinfection suggests a potential role for these cells in long-term protection. As significant progress continues to be made in the field of HuNoV VLP vaccines, it is important to determine the breadth of the immune responses to vaccination. Of particular interest is the evaluation of newly discovered correlates of protection against HuNoV gastroenteritis.

This study demonstrates the induction of memory B-cell responses following intramuscular immunization with a bivalent GI.1/GII.4 VLP vaccine and confirms the findings of previous work on the generation of ASCs in response to vaccination. As was seen with previous studies evaluating immune responses to the bivalent vaccine, the magnitude of the responses generated to the GI.1 VLP component of the vaccine was greater than that of the responses generated to the GII.4 VLP component (16, 19, 21). ASC responses are biased toward IgA, and memory B-cell responses are biased toward IgG. In the context of immune correlates of protection, the most important finding of this study is that antigen-specific IgG memory B cells persisted at day 180 postvaccination for both GI.1 and GII.4 VLPs. The concentration of GI.1 VLP-specific IgG memory B cells is very similar to that of memory B cells generated in response to GI.1 infection. The B-cell responses also correlated with serum antibody levels at several time points. ASC levels at day 7 postvaccination correlated with serum antibody levels at day 21, while the memory B-cell titers at day 56 correlated with the antibody titers at 6 and 12 months after vaccination. Memory B-cell titers also correlated significantly with prevaccination HBGA-blocking antibody titers, suggesting a role of memory B-cell populations in long-term immunity.

Intramuscular immunization with GI.1/GII.4 VLPs induced an IgG memory B-cell response stronger than that reported for intranasal immunization with 50 μg or 100 μg GI.1 VLPs adjuvanted with monophosphoryl lipid A (MPL) and chitosan. Following intranasal immunization, the percentage of GI.1-specific IgG memory B cells peaked at day 56 postvaccination and declined by day 180 (20). Although identical methods were not used to evaluate memory B cells in these studies, the percentage of IgG memory B cells was higher following intramuscular immunization and persisted for a longer duration of time.

The overall kinetics of the ASC responses following vaccination were similar to what has been described previously following infection and vaccination. ASC responses peaked at day 7 postvaccination, as was observed in infection studies (18). The dramatic decline in ASC levels by day 28 after the first dose of vaccine and a minimal increase after a second dose of vaccine were also seen and were described previously when ASC assays were carried out with fresh PBMCs (19). Although a direct head-to-head comparison of the ASC responses obtained with frozen and fresh PBMCs (the latter was expressed as counts per million CD19^+^ cells) was not possible, a strong correlation between the overall results was seen, suggesting the suitability of both fresh and frozen cells for the evaluation of HuNoV-specific ASCs. This may be particularly important in the context of field studies, where it may not be feasible to test fresh PBMC samples.

The magnitudes of the B-cell responses to NV infection and vaccination were compared. Vaccination induced peak ASC responses and IgG memory B-cell responses at days 28 and 180 comparable to those induced by NV infection, and vaccination induced an IgA memory B-cell response at day 180 lower than that induced by NV infection. Interestingly, while the memory B-cell response to infection peaked at day 14, no peak time point for vaccination-induced IgA and IgG memory B cells was identified. Data were not obtained at the day 14 time point in the vaccine studies, but the peak responses varied between vaccine groups and between IgA and IgG assays. Some of these differences may be due to the lower number of persons included in the memory B-cell analysis. No difference in the B-cell responses between secretors and nonsecretors was seen, as has been reported previously by the use of serology (data not shown).

Overall, the study demonstrates the induction of B-cell responses to intramuscular immunization with a bivalent HuNoV vaccine. While vaccination induces the production of IgG memory B cells, it remains to be evaluated if these responses also function as vaccine-induced correlates of protection. B-cell responses correlate with serum antibody responses, including HBGA-blocking antibody and serum IgA responses, which were identified to be correlates of protection in previous vaccine studies. The relative importance of these different immune markers and how they covary will need to be ascertained in future studies.

## MATERIALS AND METHODS

### Clinical study design.

A phase I, randomized, multisite, dose-escalation study of the safety and immunogenicity of intramuscular immunization with a bivalent HuNoV VLP vaccine candidate was carried out as described previously (16). Briefly, bivalent vaccine formulations contained escalating doses (5 μg, 15 μg, 50 μg, or 150 μg) of both GI.1 and GII.4 (consensus) VLPs and were adjuvanted with 50 μg of monophosphoryl lipid A (MPL) and 0.5 mg of aluminum hydroxide [Al(OH)_3_] (Table 5). The GI.1 VLP was based on the sequence of the major capsid protein VP1 of Norwalk virus, while the GII.4 VLP was designed on the basis of a consensus sequence obtained by aligning VP1 sequences from three GII.4 variants, and both were produced by Takeda Vaccines, Inc. (24). Healthy, adult participants 18 to 49 years of age were administered 2 doses of the vaccine or placebo 28 days apart by the intramuscular route. A maximum of 10 persons receiving vaccine and 2 receiving placebo were included in each vaccine dose group. The studies (registration no. NCT01168401 at ClinicalTrials.gov) were carried out at the University of Rochester and the Saint Louis University Medical Center and were approved by the institutional review board at each institution.

**TABLE 5:** Composition of placebo and GI.1/GII.4 bivalent VLP vaccine formulations per 0.5 ml

Group	No. of participants	Amt of GI.1 VLPs (μg)	Amt of GII.4 VLPs (μg)	Total amt of VLPs/dose (μg)	Amt of MPL (μg)	Amt of Al(OH)_3_ (mg)
Placebo	8	0	0	0	0	0
5/5 μg VLP vaccine	10	5	5	10	50	0.5
15/15 μg VLP vaccine	9	15	15	30	50	0.5
50/50 μg VLP vaccine	9	50	50	100	50	0.5
150/150 μg VLP vaccine	6	150	150	300	50	0.5

### Measurement of IgA and IgG ASCs and memory B cells.

Peripheral blood mononuclear cells (PBMCs) were collected from study persons prior to (days 0 and 28) and 7 days after each vaccine dose (days 7 and 35) for measurement of ASCs. Cryopreserved PBMCS were used for determining the number of ASCs by enzyme-linked immunosorbent spot (ELISPOT) assays as described previously (18). Briefly, 96-well polyvinylidene difluoride (PVDF) membrane plates (Millipore) were coated with 1 μg per well of GI.1 or GII.4 (consensus) VLPs or phosphate-buffered saline (PBS) overnight at 4°C. Cryopreserved PBMCs were thawed, and doubling dilutions were prepared in AIM-V medium (Invitrogen) supplemented with 10% heat-inactivated fetal bovine serum (Invitrogen), sodium pyruvate (Invitrogen), minimum nonessential amino acids (Invitrogen), and β-mercaptoethanol (Invitrogen), starting with 5 × 10^5^ PBMCs per well in the first row. Following incubation in a humidified incubator with 5% CO_2_ at 37°C for about 18 h, IgA and IgG ASCs were detected using goat anti-human IgA and IgG antibodies conjugated to horseradish peroxidase (HRP), respectively (Southern Biotech), and stable diaminobenzidine (DAB) as a substrate (Invitrogen). The membrane was allowed to dry overnight, and spots were imaged using an ImmunoSpot reader (Cellular Technology Limited).

### Measurement of total and VLP-specific IgA and IgG memory B cells.

Cryopreserved PBMCs collected prior to each vaccine dose (days 0 and 28) and at days 56 and 180 after the first vaccination were used for memory B-cell studies. ELISPOT assays for the measurement of total and VLP-specific IgA and IgG memory B cells were carried out as described previously (18). Briefly, cryopreserved PBMCs were thawed and expanded in supplemented AIM-V medium (Invitrogen) containing 1:100,000 pokeweed mitogen (Sigma), 1:10,000 Staphylococcus aureus Cowan strain (Sigma), and 1.25 μg/ml CpG-2006 DNA (InvivoGen) for 6 days. To determine the number of total IgA and IgG memory B cells, 96-well PVDF membrane plates (Millipore) were coated with 10 μg per well of anti-human IgA monoclonal antibody (Sigma) or anti-human IgG antibody (Jackson ImmunoResearch), respectively. PBS was used as a negative control. For VLP-specific memory B cells, the plates were coated with 10 μg per well of GI.1 or GII.4 (consensus) VLPs or PBS as a negative control. PBMCs were harvested after 6 days, and doubling dilutions of the cells were prepared such that there were 5 × 10^4^ PBMCs per well in the first dilution for the total memory B-cell assays and 5 × 10^5^ PBMCs per well in the first dilution for the VLP-specific assays. The plates were incubated in a humidified incubator with 5% CO_2_ at 37°C for about 6 h. IgA and IgG memory B cells were detected using goat anti-human IgA and IgG antibodies conjugated to HRP (Southern Biotech), respectively, overnight at 4°C, followed by the addition of stable DAB substrate (Invitrogen). Spots were imaged as described above for the ASC assays.

### Data analysis.

Dilutions of PBMCs that gave between 8 and 200 spots were used for calculation of the final results. The limit of detection was set at 8 cells per 5 × 10^5^ PBMCs for both assays. To allow comparison with previously published results on cellular immune responses to HuNoV infection (18), IgA and IgG ASC results were expressed as the number of ASCs per million PBMCs, while memory B-cell assay results were expressed as the percentage of VLP-specific memory B cells per 5 × 10^5^ total memory B cells. The limits of detection for each of the assays were therefore 16 ASCs per million PBMCs, 80 total memory B cells per 5 × 10^5^ PBMCs, and 8 VLP-specific memory B cells per 5 × 10^5^ PBMCs. For all samples with spot counts below the limit of detection of the assay, a value of one-half of the limit of detection of that assay was assigned and used for calculating the geometric mean titers and fold rises. Samples with total memory B cells below the limit of detection were not included for calculation of the percentage of VLP-specific memory B cells in the final analysis.

Serological responses, including the titers of total immunoglobulin (pan-Ig), IgA, IgG, and HBGA-blocking antibodies to GI.1 and GII.4 (consensus) VLPs, for this clinical study have been published previously (16). The correlations between B-cell responses and serological responses at various time points were assessed using Spearman correlation coefficients. Paired *t* tests were used for comparison of the responses between GI.1 and GII.4 VLPs, as well as those between the IgA and IgG assays. For study participants enrolled at the University of Rochester site, titers of IgA and IgG ASCs were previously determined using fresh PBMCs (19) and were compared to results obtained using cryopreserved PBMCs in the present study, also using Spearman correlation coefficients. In addition, the cellular immune response to the GI.1 VLP component of the vaccine that was generated was compared to previously published results on the cellular immune response following oral inoculation with GI.1 virus (18). No adjustments were made for multiple comparisons.

## Supplementary Material

Supplemental material
