# Supplementary material for: B-Cell Responses to Intramuscular Administration of a Bivalent Virus-Like Particle Human Norovirus Vaccine
Source: Clin Vaccine Immunol. 2017 May 5;24(5):e00571-16. doi: 10.1128/CVI.00571-16 (PMC5424242; doi:10.1128/CVI.00571-16)
Supplement: Supplemental material [file supp_24_5_e00571-16__index.html]

B-Cell Responses to Intramuscular Administration of a Bivalent Virus-Like Particle Human Norovirus Vaccine — Supplemental material 

# B-Cell Responses to Intramuscular Administration of a Bivalent Virus-Like Particle Human Norovirus Vaccine

## Supplemental material

- Supplemental file 1 -

  Fig. S1. Kinetics of antibody-secreting cell (ASC) responses to GI.1 and GII.4 (consensus) VLPs.

  PDF, 149K
- Supplemental file 2 -

  Fig. S2. Comparison of antibody-secreting cell (ASC) responses to GI.1 and GII.4 (consensus) VLPs at day 7 after vaccine dose 1.

  PDF, 25K
- Supplemental file 3 -

  Fig. S3. Kinetics of memory B-cell responses to GI.1 and GII.4 (consensus) VLPs.

  PDF, 280K
- Supplemental file 4 -

  Fig. S4. Comparison of memory B-cell responses to GI.1 and GII.4 (consensus) VLPs.

  PDF, 36K
