# Supplementary material for: B-Cell Responses to Intramuscular Administration of a Bivalent Virus-Like Particle Human Norovirus Vaccine
Source: Clin Vaccine Immunol. 2017 May 5;24(5):e00571-16. doi: 10.1128/CVI.00571-16 (PMC5424242; doi:10.1128/CVI.00571-16)

Supplementary Figure 1: Kinetics of antibody secreting cell (ASC) responses to GI.1 and GII.4 (consensus) VLPs. Geometric mean numbers of ASCs per million peripheral blood mononuclear cells (PBMCs) produced in response to intramuscular immunization with different doses of GI.1 and GII.4 VLPs are shown. IgA ASC responses for GI.1 and GII.4 VLPs are given in panels A and B, respectively, while IgG responses are shown in panels C and D. Error bars represent 95% confidence intervals.

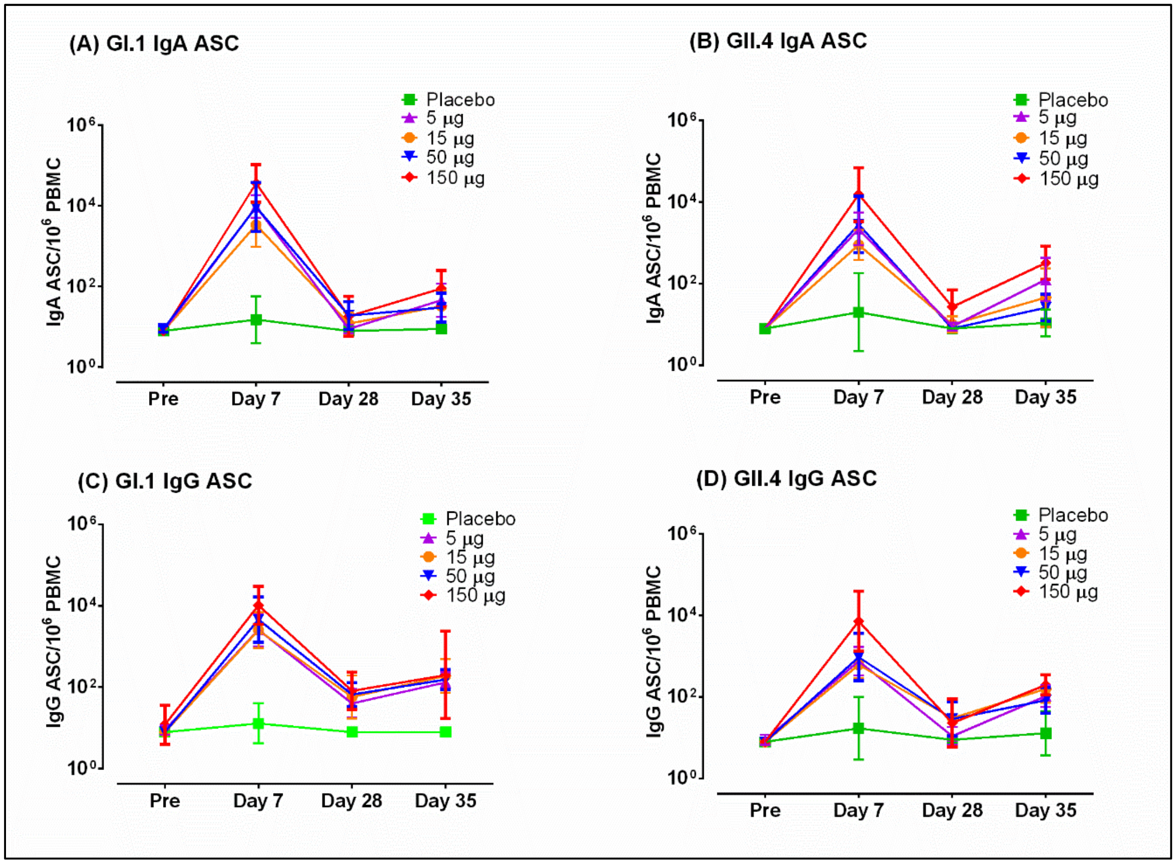

Supplement: Supplemental material [file CVI.00571-16_zcd999095466s1.pdf]
