# Supplementary material for: B-Cell Responses to Intramuscular Administration of a Bivalent Virus-Like Particle Human Norovirus Vaccine
Source: Clin Vaccine Immunol. 2017 May 5;24(5):e00571-16. doi: 10.1128/CVI.00571-16 (PMC5424242; doi:10.1128/CVI.00571-16)

Supplementary Figure 2: Comparison of antibody secreting cell (ASC) responses to GI.1 and GII.4 (consensus) VLPs at day 7 post vaccine dose 1. Peak IgA ASC (a) and IgG ASC (b) responses to intramuscular immunization with the GI.1 and GII.4 VLP formulation were seen at day 7 post first dose of vaccine. Overall, the magnitude of responses to GI.1 VLPs was higher than to GII.4 VLPs ($p<0.001$). Each bars represents the geometric mean of results for that group (error bars, 95% confidence intervals)

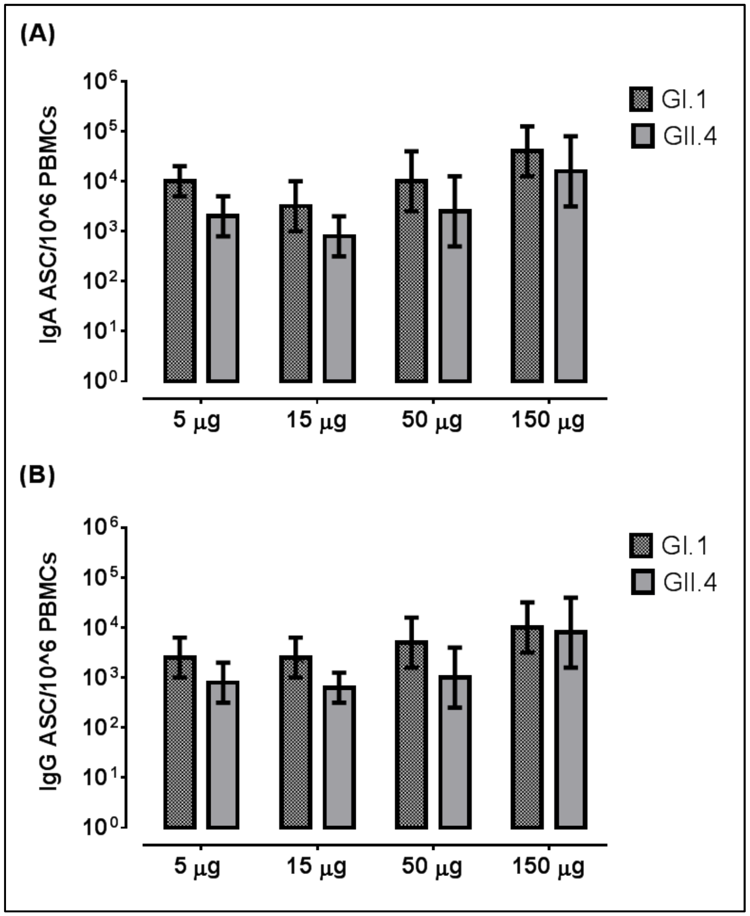

Supplement: Supplemental material [file CVI.00571-16_zcd999095466s2.pdf]
