# Supplementary material for: B-Cell Responses to Intramuscular Administration of a Bivalent Virus-Like Particle Human Norovirus Vaccine
Source: Clin Vaccine Immunol. 2017 May 5;24(5):e00571-16. doi: 10.1128/CVI.00571-16 (PMC5424242; doi:10.1128/CVI.00571-16)

Supplementary Figure 3: Kinetics of memory B-cell responses to GI.1 and GII.4 (consensus) VLPs. Geometric mean numbers of VLP-specific memory B-cells per 5 X 10^5 total memory B-cells produced in response to intramuscular immunization with different doses of GI.1 and GII.4 VLPs are shown. IgA memory B-cell responses for GI.1 and GII.4 VLPs are given in panels A and B, respectively, while IgG responses are shown in panels C and D. Error bars represent 95% confidence intervals.

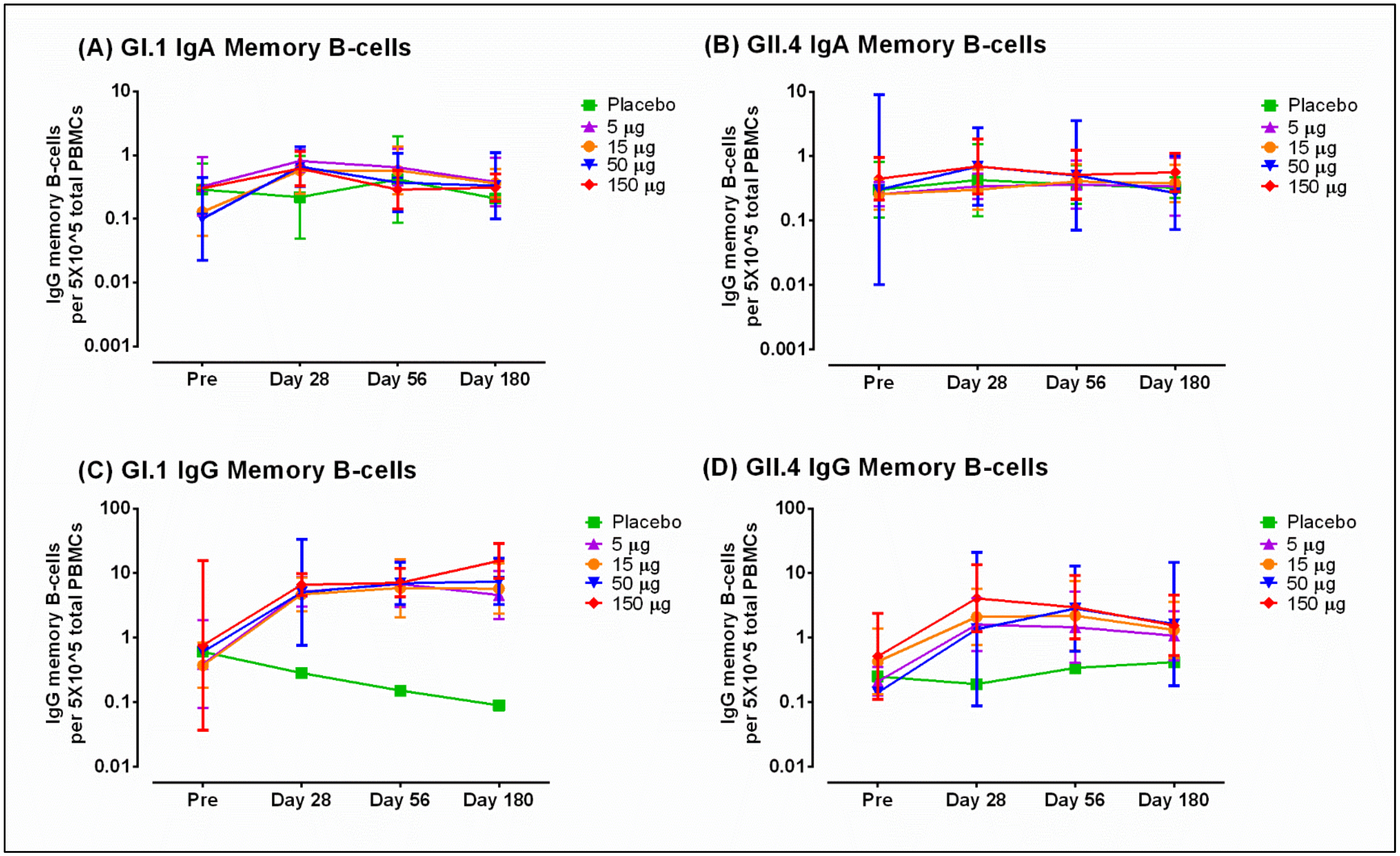

Supplement: Supplemental material [file CVI.00571-16_zcd999095466s3.pdf]
