# Supplementary material for: B-Cell Responses to Intramuscular Administration of a Bivalent Virus-Like Particle Human Norovirus Vaccine
Source: Clin Vaccine Immunol. 2017 May 5;24(5):e00571-16. doi: 10.1128/CVI.00571-16 (PMC5424242; doi:10.1128/CVI.00571-16)

Supplementary Figure 4: Comparison of memory B-cell responses to GI.1 and GII.4 (consensus) VLPs. IgA memory B-cells at days 28 and 56 post-vaccination (A, B) and IgG memory B-cell responses at days 28 and 56 post-vaccination (C, D) following intramuscular immunization with the GI.1 and GII.4 VLP formulation. Overall, the magnitude of IgA memory B-cell response to GI.1 VLP was significantly higher than to GII.4 VLPs at day 28 ($p=0.03$) but not day 56. IgG memory B-cell responses to GI.1 VLP were significantly higher than responses to GII.4 VLPs at both days 28 and 56 post vaccination ($p<0.05$). Each bar represents the geometric mean of results for that group (error bars 95% confidence intervals)

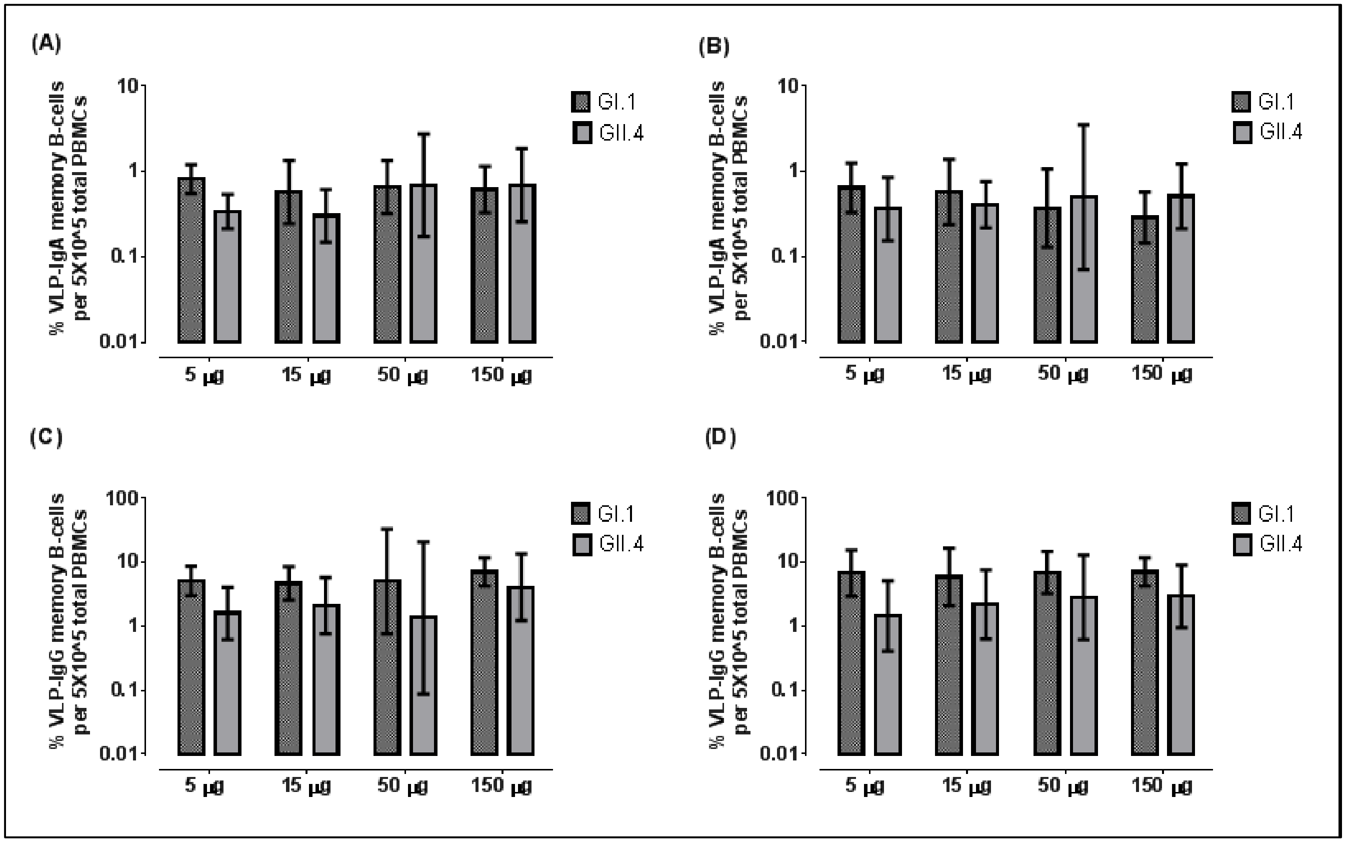

Supplement: Supplemental material [file CVI.00571-16_zcd999095466s4.pdf]
